# Body mass index and trajectories of the cognition among Chinese middle and old-aged adults

**DOI:** 10.1186/s12877-022-03301-2

**Published:** 2022-07-23

**Authors:** Wei Zhang, Yanan Chen, Na Chen

**Affiliations:** 1 School of Health Economics and Management, Nanjing University of Chinese Medicine, Nanjing, Jiangsu, People’s Republic of China; 2 School of Elderly Care Services and Management, Nanjing University of Chinese Medicine, 138 Xianlin Road, Nanjing, Jiangsu, People’s Republic of China

**Keywords:** Body mass index, Cognitive decline, Group-based trajectory modeling, CHARLS

## Abstract

This study aims to investigate the association between trajectories of the cognition and body mass index (BMI) among Chinese middle and old-aged adults. A total of 5693 adults (age 45 +) whose cognitive score is higher than average at the baseline were included from China Health and Retirement Longitudinal Study (CHARLS:2011–2015). Cognitive function was measured by Mini-mental state examination (MMSE) in Chinese version. The Group-based trajectory modeling (GBTM) was adopted to identify the potential heterogeneity of longitudinal changes over the past 5 years and to investigate the relationship between baseline BMI and trajectories of cognitive function. Three trajectories were identified in results: the slow decline (37.92%), the rapid decline (6.71%) and the stable function (55.37%). After controlling for other variables, underweight (BMI < 18.5 kg/m^2^) was associated with the rapid and slow decline trajectories. Obesity (BMI > 28 kg/m^2^) was associated with the slow decline trajectory. High-risk people of cognitive decline can be screened by measuring BMI.

## Introduction

With the population aging, the research on cognitive impairment has been considered as a priority. Because cognitive impairment is closely related to dementia, which is the fifth leading cause of death globally and the troublesome disease with a heavy burden on both individuals and the society [1]. In addition, it is estimated that the number of dementia patients in China will increase to 30 million by 2050 [2]. Meanwhile, due to limited successful treatments of dementia and no effective medication for cognitive impairment [3], screening potential high-risk groups and effective prevention strategies are extremely important [4].

Research found that there was a significant association between BMI and cognitive impairment [5, 6]. However, the results were controversial due to different physiological characteristics. According to previous studies, it was found that being overweight but not obesity was associated with better cognitive function in Colombia, while there was no significant correlation between BMI and cognition in Korea[7] and Caucasia [8]. It is also reported that higher BMI had adverse effects on cognitive impairment among older adults (age 65 +) in Spain [9]. Moreover, some studies have found that lower BMI was independently associated with the risk of moderate and severe cognitive impairment [10–12]. Furthermore, In the studies in China, some scholars found that higher BMI was a protective factor for cognitive function of older adults in the community [13]. When BMI was treated as a continuous variable, it was reported that the relationship between BMI and the risk of mild cognitive impairment was in a ‘U’ shape [14], indicating that there might be a more complex relationship between BMI and cognitive function in China too. However, most of the previous studies were cross-sectional and examining the relationship between BMI and cognitive risk. Our study explored the relationship between BMI at baseline and cognitive trajectory, and attempted to use BMI as an observation variable to provide an effective and convenient screening method for high-risk groups with cognitive impairment.

Previous studies have shown that there are differences in cognitive trajectories among different countries. There were two cognitive trajectories in Korean people over 60 years old [15]. Four different cognitive trajectories were found in Chinese people over 80 years old [16]. Three different cognitive trajectories were found in adults over 55 years old in Spain, and the influencing factors were different in different trajectories [17].There were three trajectories of cognition (rapid cognitive decline, slow decline and stable) among older adults (age 65 +) in mainland China [18]. However, cognitive decline may have occurred earlier, the decline of cognitive function was closely related to the status of participants in their middle age [19]. Therefore, we used a relatively large national sample to include middle-aged people over the age of 45 to observe whether there is a similar cognitive decline in this population, so that we can take action as soon as possible.

## Methods

### Sample

Data were obtained from China Health and Retirement Longitudinal Study (CHARLS) [20], a nationally representative survey of Chinese adults over 45 years. The national baseline survey was conducted in 2011, and the respondents were followed up in 2013, 2015, and 2018. However, the 2018 Mini-Mental State Examination (MMSE) score was not included because the questions on orientation and attention were changed in the 2018 cognitive assessment. Therefore, this study included data starting from 2011 (wave 1) with 17,708 respondents, 20,776 in 2013 (wave 2), to 23,470 in 2015 (wave 3). According to the purpose of this study, we formulated the inclusion criteria for subjects as follows: age ≥ 45, sex, years of schooling, marital status, medical insurance, social activities, chronic diseases, physical activities, sleeping time, and their MMSE scores results. Based on these inclusion criteria, 5693 respondents were selected. Ethical approval for all the CHARLS waves was granted from the Institutional Review Board at Peking University. The IRB approval number for the main household survey, including anthropometrics, was IRB00001052-11,015; the IRB approval number for biomarker collection, was IRB00001052-11,014.

### Cognitive assessment

The MMSE is widely used to assess cognitive function worldwide [21, 22]. The total cognition score ranges from 0 to 21, which includes the following items: the Telephone Interview of Cognitive Status (TICS-10) (orientation and attention, 5), word recall (episodic memory, 10), serial subtraction of 7 from 100(up to 5 times, 5), and figure drawing (visual spatial abilities, 1) [23]. In order to better observe the cognitive decline, individuals with above-average cognitive scores at baseline were included in the study [24]. The investigators of China Health and Retirement Longitudinal Study performed the MMSE in each Wave, all of them were professionally trained to reduce systematic difference as much as possible.

### Body mass index

Information about weight and height was collected at baseline as well as follow-up interviews. BMI was calculated by body weight (kg) divided by square of height (m^2^), height and weight comes from on-site measurements. Globally, the average BMI varies greatly among different countries [25, 26]. Using a lower BMI threshold than the WHO international standard or the improved Asian BMI standard to define overweight and obesity can better illustrate the current body mass index status of Chinese people [27, 28]. Refer to the Chinese criteria for adults, BMI categories were defined as underweight (< 18.5 kg/m^2^), normal weight (18.5–23.9 kg/m^2^), overweight (24–27.9 kg/m^2^), and obesity (≥ 28 kg/m^2^) [16].

### Covariates

We included the following variables as covariates in this research: age, gender (1 = male, 2 = female), marital status (1 = married, 0 = unmarried), years of schooling, medical insurance (1 = have medical insurance, 0 = otherwise), social activities (1 = more than once a week, 0 = otherwise), chronic diseases(0 = none, 1 = less than three diseases, 2 = more than three diseases), physical activities(1 = more than once a week, 0 = otherwise), and sleeping time [29–31].

### Data analysis

Trajectories of MMSE score were identified by Group based trajectory modeling (GBTM), a potential class growth model used to analyse the longitudinal data and explore the heterogeneity. The principle is to assume that there is heterogeneity, that is, there are several potential subgroups with different development trajectories or patterns in the population, and its purpose is to explore how many subgroups with different development trends are included in the population, and to determine the development trajectory of each subgroup[32]..

Finding the most suitable GBTM is an iterative process, including determining the optimal number of trajectory groups and identifying the structure of their trajectory groups. Many factors need to be considered in model selection, including statistical measures (the *p-*values of model parameters and the confidence intervals of trajectory estimates), visual inspection of predicted trajectories, Bayesian Information Criterion (a smaller BIC indicating a better model fit) and the average posterior probability (AvePP; above 0.7 indicated optimal fit).

The Stata Traj plug-in (Stata Corp., College Station, TX) was used to model the cognitive trajectories with a censored normal distribution [33]. The two-way linear prediction plots were also completed by Stata. All the statistical analyses were performed using Stata 15.1. Continuous variables were presented as mean (SD), while categorical variables were presented as percentages. χ2 test or analysis of variance F-test was used to compare the data between different trajectory groups. Multivariate logit model was used to analyse the relationship between baseline BMI and trajectory of MMSE scores. The results were considered significant when *P* < 0.05.

## Results

### Descriptive characteristics

At baseline, a total of 5693 middle and old-aged adults including 3042 men and 2651 women aged 45–90 years (mean age 57.07 years) were enrolled in this research. The mean MMSE score was 13.63 out of 21 (SD = 2.18). According to the Chinese criteria, 1352 (23.75%) participants were classified as obesity, 1596 (27.93%) participants were overweight, and 211 (3.71%) participants were underweight. Most participants were married. Nearly 90% of the participants received formal education, but less than half received more than six years of formal education. Few participants (5%) did not have medical insurance. Among the participants, 40.70% had social activities at least once a week. And 34.55% of participants did not suffer from chronic diseases. Nearly half of the participants did physical activities each week. The average sleeping time is 6.50 h (SD = 1.65). Baseline characteristics of participants are presented in Table 1.

**Table 1:** Baseline characteristics of the total sample and the sample by the different trajectory groups^a^

		TRAJECTORY GROUP			
CHARACTERISTIC	TOTAL SAMPLE (*n* = 5693)	RAPID DECLINE (*n* = 361)	SLOW DECLINE (*n* = 2101)	STABLE (3231)	*P*^b^
BMI Underweight (< 18.5) Normal (18.5–23.9) Overweight (24–27.9) Obesity (> = 28) Age	211(3.71) 2540(44.62) 1596(27.93) 1352(23.75)	28(7.76) 171(47.37) 83(22.99) 79(21.88)	105(5.00) 1021(48.60) 564(26.84) 411(19.56)	78(2.41) 1348(41.72) 943(29.19) 862(26.68)	** < 0.001**
mean(SD)^c^	57.07(8.16)	60.91(9.76)	58.29(8.11)	55.85(7.73)	** < 0.001**
45–54	2340(41.10)	96(26.59)	729(34.70)	1515(46.89)	** < 0.001**
55–64	2303(40.45)	148(41.00)	890(42.36)	1265(39.15)	
65–74	901(15.83)	77(21.33)	420(19.99)	404(12.50)	
75–84	149(2.62)	40(11.08)	62(2.95)	47(1.45)	
Male	3042(53.44)	145(40.28)	1075(51.17)	1822(56.39)	** < 0.001**
Married	5243(92.10)	308(85.32)	1918(91.29)	3017(93.38)	** < 0.001**
Years of schooling					** < 0.001**
0	612(10.75)	143(39.61)	371(17.66)	98(3.03)	
1–6	2465(43.30)	151(41.83)	1163(55.35)	1151(35.62)	
> = 7	2616(45.95)	67(18.56)	567(26.99)	1982(61.34)	
Medical insurance	5410(95.0)	337(93.35)	1996(95.00)	3077(95.23)	0.295
Social activities	2317(40.70)	145(40.17)	720(34.27)	1452(44.92)	**< 0.001**
Chronic diseases					0.052
none	1967(34.55)	123(34.07)	676(32.18)	1168(36.15)	
1–3	3317(58.26)	215(59.56)	1266(60.26)	1836(56.82)	
> 3	409(7.18)	23(6.03)	159(7.57)	227(7.03)	
Physical activities	2519(44.25)	161(44.60)	880(41.88)	1478(45.74)	**0.021**
Sleeping time, mean(SD)^c^	6.50(1.65)	6.14(1.90)	6.39(1.76)	6.61(1.53)	**< 0.001**
MMSE score, mean(SD)^c^	13.63(2.18)	12.29(2.01)	12.20(1.63)	14.71(1.88)	**< 0.001**

^a^Data are presented as counts (percentage) unless otherwise indicated

^b^P value determined using χ2 test or analysis of variance F-test

^c^For continuous variables, mean (SD) for each trajectory group and significance from analysis of variance F-test are reported

### Cognitive trajectory modeling

The fitting process was carried out from high-order single group to low-order multi-group (Table 2). The 4-group has the best Bayesian Information Criterion (2ΔBIC), the AvePP values are all more than 0.8. The proportion of subgroups within the group were > 5%. It was considered that the fourth group is the best model.

**Table 2:** AvePP of Group Assignment and BIC Statistics of Model Fit

No	Order	2ΔBIC	Proportion	AvePP
1	2	-	100%	100%
2	2,2	3308.84	23.07%/76.93%	88.66%/95.37%
3	2,2,2	1109.68	5.51%/35.36%/59.13%	90.10%/84.69%/90.30%
3(1)	2,2,2,2	781.7	1.81%/11.38%/46.12%/40.70%	95.46%/86.42%/82.08%/86.24%
**4**	**1,2,2**	**15.46**	**6.71%/37.92%/55.37%**	**90.63%/84.08%/88.91%**
5	1,1,2	64.16	7.30%/39.80%/52.90%	90.64%/83.63%/88.18%

**Fig. 1:**
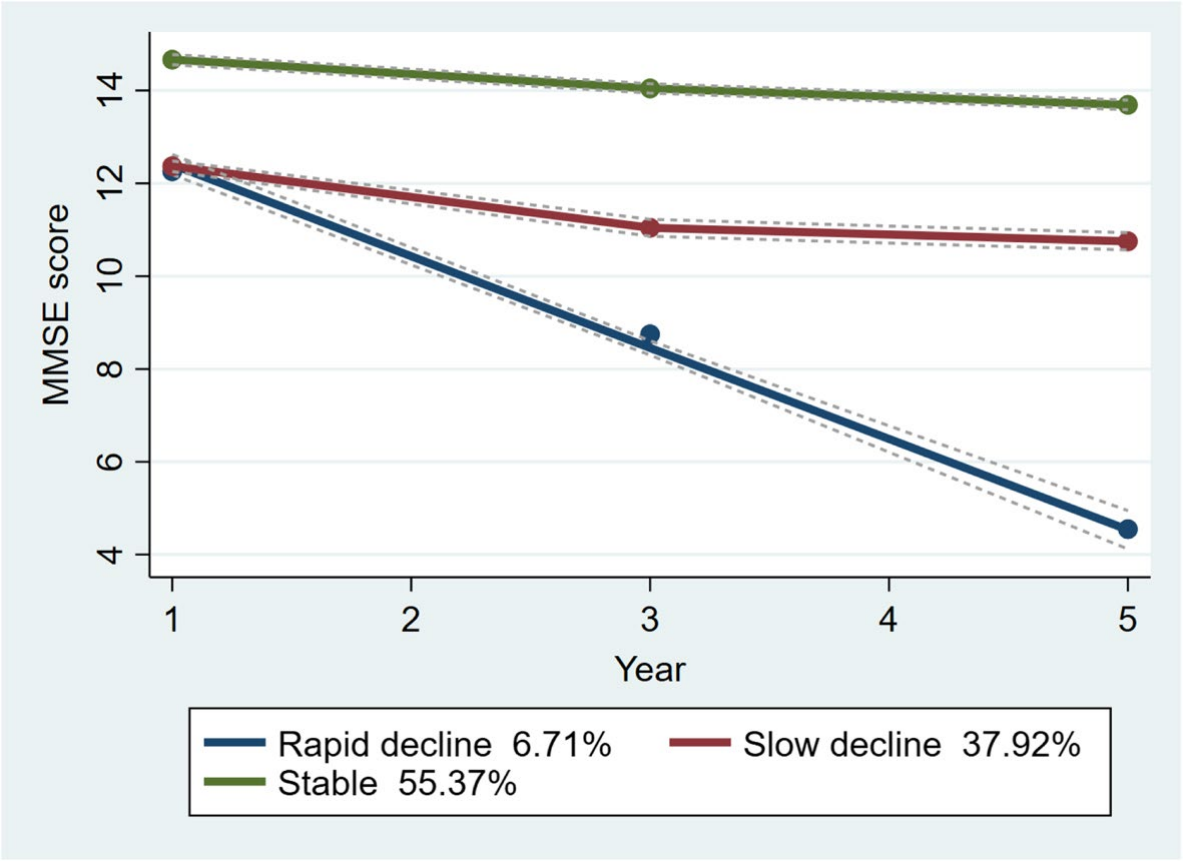
Trajectories of the MMSE scores. The solid lines (green: stable red: slow decline blue: decline) mean estimated values, and the dotted lines display the 95% CIs

Therefore, three groups were identified in the three-phase follow-up data (Fig. 1). (1) One group showed a stable and higher MMSE score (STABLE FUNCTION, 55.37%). The stable group was characterized by the fact that the MMSE score of three stages was basically unchanged from 14.71 to 13.75. (2) One group had a lower MMSE score in the baseline and then maintained a slow decline (SLOW DECLINE, 37.92%). The slow decline group was characterized by a steady decline in the MMSE score from 12.20 in the baseline to 10.54. (3) One group decreased rapidly throughout the process (RAPID DECLINE, 6.71%). The decreasing trend was very obvious in the rapid decline group, and the MMSE score decreased from 12.29 to 4.24 in 5 years.

As shown in Table 1, there was a significant relationship between BMI and trajectory groups (*P* < 0.01). Furthermore, there were significant differences among age, gender, marital status, years of schooling, social activities and MMSE scores and trajectory groups. Compared with the stable group, the average age of the slow decline group was slightly older (*M* = 58.29, *SD* = 8.11), and the figure in the rapid decline group was 60.91(*SD* = 9.76). The proportion of male and married adults (age 45 +) in the rapid decline group was slightly lower, but the number of people with lower years of schooling was larger. The baseline MMSE score of the stable group was the highest (*M* = 14.71, *SD* = 1.88), while the scores were similar in the slow decline group (*M* = 12.20, *SD* = 1.63) and the rapid decline trajectory group (*M* = 12.29, *SD* = 2.01).

### Relationship between BMI and cognitive trajectories

Univariate logit regression analysis showed that compared with the stable group, the odds ratio of underweight in the rapid decline group was 2.830 and that in the slow decline group was 1.777. After adjusting for some demographic factors and healthy factors (age, gender, marital status, years of schooling, medical insurance, social activities, chronic diseases, physical activities, sleeping time), the correlation was still significant (rapid decline group: *OR* = 2.238, 95%*CI* = 1.346–3.721, slow decline group: *OR* = 1.523, 95%*CI* = 1.098–2.113). Compared with the stable group, obesity was significantly correlated with the decline of cognitive function in the slow decline group (*OR* = 0.695, 95%*CI* = 0.595–0.813) after demographic factors and healthy factors (Table 3).

**Table 3:** Association of body mass index with trajectories of MMSE scores

RAPID DECLINE vs STABLE				SLOW DECLINE vs STABLE		
Adjustment	OR	95%CI	*P*	OR	95%CI	*P*
Model 1						
Normal (18.5 ~ 23.9)	1			1		
Underweight (< 18.5)	2.830	1.786,4.483	** < 0.001**	1.777	1.311,2.409	** < 0.001**
Overweight (24 ~ 27.9)	0.694	0.527,0.914	**0.009**	0.790	0.692,0.901	** < 0.001**
Obesity (≥ 28)	0.722	0.546,0.956	**0.023**	0.630	0.546,0.726	** < 0.001**
Model 2						
Normal (18.5 ~ 23.9)	1			1		
Underweight (< 18.5)	2.315	1.397,3.837	**0.001**	1.597	1.154,2.210	**0.005**
Overweight (24 ~ 27.9)	0.782	0.582,1.051	0.103	0.857	0.743,9,989	**0.034**
Obesity (≥ 28)	0.801	0.593,1.108	0.150	0.684	0.586,0.798	** < 0.001**
Model 3						
Normal	1			1		
Underweight (< 18.5)	2.238	1.346,3.721	**0.002**	1.523	1.098,2.113	**0.012**
Overweight (24 ~ 27.9)	0.821	0.610,1.105	0.193	0.870	0.753,1.005	0.059
Obesity (≥ 28)	0.827	0.610,1.123	0.224	0.695	0.595,0.813	** < 0.001**

*Note*: model 1: no adjustment; model 2: adjust for age, gender, marital status, years of schooling, medical insurance; model 3: adjust for age, gender, marital status, years of schooling, medical insurance, social activities, chronic diseases, physical activities, sleeping time

## Discussion

In this study, we investigated cognitive trajectories of a nationally representative sample of 5693 Chinese middle and old-aged adults (age 45 +) who were followed up for three waves; we also explored the relationship between baseline BMI and cognitive trajectories. Our results showed that there were three different trajectories of cognitive function, namely, stable function, slow decline, and rapid decline. Different BMI statuses are associated with different cognitive trajectories.

Our finding demonstrated that cognitive function declines with age, which was consistent with previous studies [18]. Furthermore, the majority of participants have the stable trajectory, and rapid decline of cognitive function was rare among the respondents. This finding is consistent with previous studies [15, 34].

BMI < 18.5 kg/m^2^ was associated with an OR of 2.238 and 1.523 for participants in the rapid and slow decline groups compared to stable one, respectively. This finding supported that BMI < 18.5 kg/m^2^ was a risk factor for cognitive decline, which is in line with previous studies [35]. Underweight may be related to sarcopenia, which was considered as a potential risk factor for cognitive impairment [36]. Lower BMI may indicate malnutrition, and nutritional status is closely related to cognitive function via biological mechanisms [12]. At the same time, weight loss may be a precursor to dementia. Patients with Alzheimer's disease may have pathological changes in their olfactory nerves, reduced attractiveness of food and food intake, resulting in weight loss [37].

BMI ≥ 28 kg/m^2^ (OR = 0.695) was protective factor for slow cognitive decline, although the OR in rapid decline group were not significant. Similar results have been found in cohort studies in South Korea and Singapore, which supports the "obesity paradox" [38, 39]. The study of Forte (2017) showed that maintaining a certain proportion of gynoid fat can put off the cognitive decline [40]. According to the guidelines in the United States, a variety of physical activities such as balance training, aerobics and muscle strengthening can reduce the risk of cognitive decline [41]. People with higher BMI at the basic stage may try to lose weight because of health issues [42]. The risk of cognitive decline among these people may be reduced due to physical exercise, as it is a common way to lose weight.

What is worth exploring is that for the middle and old-aged adults (age 45 +) in China, the research on the protective effect of higher BMI on cognitive function is limited among adults age 45 + in China. In the rapid decline group, higher BMI at baseline was not a protective factor for cognitive decline. It may be because the cognitive function with rapid cognitive decline usually has undergone irreparable physiological pathological changes. Rapid cognitive decline was associated with health conditions, such as blood–brain barrier breakdown, which may interfere with the protective effect of high BMI on cognitive function [43, 44].In addition, we speculate that this may also be related to relatively small number of participants in the rapid decline group (6.7%).

According to our research, cognitive decline in the Chinese population starts at least from the age of 45, in order to prevent an increasing prevalence of cognitive decline and dementia worldwide, we suggest that more attention should be paid to the BMI, which is an easily available indicator of cognitive decline, even for someone with mobility difficulties. Furthermore, we should pay more attention to middle-aged adults (age 45 +) as well as older adults (60 +). When it is found that the middle-aged adults who are underweight (BMI < 18.5 kg/m^2^), we can suggest them to improve nutrition and do more physical activities so as to alleviate the possibility of cognitive decline in future.

The study has some limitations. First of all, the criteria of MMSE scale in the fourth wave were different from those of the first three waves. In order to be consistent, we did not include the fourth wave of data. Second, there is a mixture in the measurement of weight and height in CHARLS, we have corrected most of the data, but there may still be a small margin of error. Third, although other similar studies included variables such as depressive symptoms, dietary intake, smoking and drinking, there were many missing values in the early data; therefore, we did not adopt these variables when fitting regression models. Finally, the results of our study are only for the middle and old-aged adults in China. If we want to extend the conclusions to the whole Asian region, more comprehensive data are needed.

## Conclusions

There were three trajectories of cognitive function among Chinese adults (age 45 +). Underweight (BMI < 18.5 kg/m^2^) was associated with an increased risk of cognitive decline. Moreover, obesity (BMI > 28 kg/m^2^) had a protective effect on cognitive function only in the trajectory of slow decline. Based on our findings, to identify middle-aged and older adults in the community who are underweight (BMI < 18.5 kg/m^2^) and to conduct annual BMI calculation could be a low-cost way to screen people at risk of cognitive decline.

## Data Availability

The data that support the findings of this study are available from CHARLS but restrictions apply to the availability of these data, which were used under license for the current study, and so are not publicly available. Data are however available from the authors upon reasonable request and with permission of CHARLS (https://charls.charlsdata.com/pages/data/111/zh-cn.html).

## Abbreviations

AvePP: Average posterior probability
BMI: Body mass index
BIC: Bayesian Information Criterion
CI: Confidence Intervals
CHARLS: China Health and Retirement Longitudinal Study
GBTM: Group-based trajectory modeling
IRB: Institutional Review Board
MMSE: Mini-mental state examination
OR: Odds ratio
TICS: Telephone interview for cognitive status
